# Comparison of two alcohol hand rubbing techniques regarding hand surface coverage among hospital workers: a quasi-randomized controlled trial

**DOI:** 10.1186/s13756-022-01172-1

**Published:** 2022-11-03

**Authors:** Yumi Suzuki, Motoko Morino, Ichizo Morita, Sumie Ohiro

**Affiliations:** 1 Department of Pediatrics, NHO Shimoshizu National Hospital, 934-5 Shikawatashi, Yotsukaidou-City, Chiba 284-0003 Japan; 2 Division of Infection Control, NHO Shimoshizu National Hospital, Yotsukaidou, Japan; 3 Department of Nursing, NHO Shimoshizu National Hospital, Yotsukaidou, Japan; 4 Japanese Red Cross Toyota College of Nursing, Toyota, Japan

**Keywords:** Hand hygiene, Alcohol based hand rub, Hand rubbing technique, Guideline adaptation, Hand size

## Abstract

**Background:**

The adapted 6-step without interlock (A6Sw/oI) hand rub technique, commonly practiced in Japan, adds the “wrist” but omits the “interlock” step compared to the WHO 6-step technique (WHO6S). The first objective of this study was to assess the differences of the two techniques regarding surface coverage. The second objective was to analyze the coverage differences between hand sizes.

**Methods:**

Hospital workers went under stratified quasi-randomization by glove size. The overall mean coverage, and the coverage of the sections of the hands were evaluated by fluorescent dye-based coverage assessment using a digital device with artificial intelligence technology.

**Results:**

Total of 427 workers were randomly allocated to WHO6S (N = 215) or the A6Sw/oI (N = 212). The overall mean dorsum coverage by WHO6S and A6Sw/oI was 90.6% versus 88.4% (*p* < 0.01), and the percentage of the participants with insufficient coverage of the backs of the four fingers ranged from 0.0–7.4% versus 28.2–51.4% (*p* < 0.001). Dorsum coverage varied largely between hand size for both techniques, and significant differences were found between small and large hands.

**Conclusion:**

The WHO6S was superior to the locally adapted technique regarding hand surface coverage. Hand size should be considered when assessing coverage differences between procedures.

No trial registrations or fundings.

**Supplementary Information:**

The online version contains supplementary material available at 10.1186/s13756-022-01172-1.

## Introduction

Hand hygiene (HH) plays a key role in preventing hospital-acquired infections, as it prevents the spread of infectious organisms from patient to patient through the contamination of healthcare workers’ hands [1–3]. HH by alcohol-based hand rub (ABHR) is widely recommended in Japan; however, national or subnational HH initiatives that are guideline-based or evidence-based still do not exist. Most Japanese healthcare workers (HCWs) take “one push” of ABHR and refer to the diagram shown on the website of the Japanese Ministry of Health, Labour and Welfare [4] to practice the hand rubbing procedure. This locally adapted diagram consists of 6 steps, which is the same number as that of the World Health Organization (WHO) 6-step technique (WHO6S) [5], but the difference is that the “interlock” step, with backs of fingers to opposing palms with fingers interlocked, is replaced by the “wrist” step. There is no publication to explain why and when this replacement occurred. However, this adapted 6-step without interlock technique (A6Sw/oI) is often considered to be officially recommended and is generally used to train HCWs and students in most Japanese health care and educational facilities. Not including the “interlock” step, the step to cover the backs of the fingers, is not unique to Japan; for example, a subnational programme currently presents a diagram similar to the WHO6S without the “interlock” step [6]. At a quick glance, this diagram seems to include the 6 steps of the WHO technique, and the drawing of the step to “Rub fingertips of each hand in opposite palm” may seem to look similar to the “interlock” step of the WHO6S. However, a dedicated step which clearly focuses on rubbing the backs of the four fingers, is missing. Theoretically, lacking the “interlock” step in the locally adapted techniques would lead to insufficient coverage of the backs of the four fingers. However, to date, there is no evidence that compares the two techniques. The primary objective of this study was to assess the difference in the hand surface coverage of the WHO6S and the A6Sw/oI considering hand size, using a newly developed device using artificial intelligence technology. The secondary objective was to compare the coverage differences between hand size, within each technique.

## Methods

### Trial design

A stratified parallel randomized group comparison, allocation ratio of 1:1.

### Participants

All full-time workers of the facility, including the non-HCWs, were enrolled in the study as a part of the second mandatory infection control training of the fiscal year, from November 2021 to January 2022. Workers with major anomalies and deformations and/or severe hand eczema with exudates were excluded from the data collection.

### Settings

The study was conducted at NHO Shimoshizu National Hospital, which has 440 beds, located in eastern Japan. The hospital is not a typical 'teaching hospital', except for some specialties that have limited teaching functions. The hospital has adopted the WHO HH strategy since 2014, but until 2019, the A6Sw/oI diagram was used throughout the hospital [7]. From 2020, the infection control team changed the diagram to an adapted version of the WHO6S including interlock with some minor changes (fingertips first, thumbs second) throughout the hospital. All participants had received their first mandatory infection control training on this adapted version including interlock procedure in May 2021. The participants were told that they would be randomly assigned to one of the two techniques that were both officially recommended but were both slightly different from the technique that was taught in the training session in May. It was made clear that their data will be used in a study, but the purpose of the study was not disclosed. The participants were not rewarded or punished by the results but were given feedback on the results and personal advice on their performance by looking at their coverage data. The participants each came to the infection control room separately according to the predetermined schedule, where all the data were taken by the same investigator throughout the study.

### Interventions

#### Hand rubbing procedures

One full push, 1.1 ml of fluorescent-marked formula, a 1:1 mixture of fluorescent dyed cream and gel type ABHR (Pure Rubbing, 83% ethanol with lipidure, Schuelke Japan, Tokyo, Japan), was given to each participant by an investigator. The cream and the ABHR gel were mixed in the forementioned composition ratio, to achieve both clear enough images, and acceptable texture and viscosity. The mixed substance was put back into the original 500 ml dispenser of the ABHR product, with the one push amount of 1.1 ml. Although there is a growing consensus that the amount of ABHR should be customized to hand size for hand rubbing in clinical practice, application volume was fixed in this study to clarify the effect of differences by hand size. The amount was chosen as to reflect the daily use in the facility at the time of the study; “one push, for all hand sizes”, with a common volume per push of the ABHR products currently available in Japan. The participants were requested to hand rub exactly according to their assigned technique, either (A) WHO6S or (B) A6Sw/oI, by looking at the corresponding diagram poster carefully, regardless of the hand rubbing technique in their daily practice. No instructions about the rubbing time were given, and the hand rubbing was performed along with the investigator who performed the same procedure, standing by the side and saying out loud only the key words for each of the 6 steps, approximately 4 s per step. The total rubbing time was fixed to approximately 25 s so that the personal difference in the length of time of rubbing would not affect the results. The diagrams and the keywords used are shown in the Additional files; (A) WHO6S diagram as Additional file 1, and (B) A6Sw/oI diagram, adapted and translated into English by the authors, referring to [4] which was the original Japanese diagram used in the study, as Additional file 2. In order to exclude the effect from the variation in coverage by participants’ voluntary movements by “rub hands until dry”, which is originally included in the WHO6S diagram, the covering/rubbing processes were strictly limited to the 6 steps of each technique. Although the rubbing time was standardized, drying time was not considered or measured. Individual comments or advice regarding the hand rubbing technique were not given prior to or during the rubbing process. These were given personally to the participants just after the analyzed data were obtained, looking together at the digital images and the score of their result. Therefore, no corrections were made during the rubbing procedure, and performances with differences from the diagrams were included in the study.

#### Coverage assessments

The coverage by each hand rubbing procedure was evaluated by SCORE! (Moraine Corporation, Tokyo, Japan). SCORE! is a new digital device using artificial intelligence (AI) technology that anatomically divides the hands into 72 sections and quantifies the coverage based on image analysis. This consists of a simple foldable black box, with ultraviolet (UV) lighting to place inside, and a dedicated iPhone7 with the SCORE! application installed, connected to the internet. After hand rubbing with the fluorescent-marked ABHR formula, the participants inserted their hands into the box, and the digital image of one side (the palmar or the dorsal side) of both hands were shown on the iPhone screen. When the investigator tapped on the ‘photo’ button, also shown on the screen, the images were recorded by the camera of the iPhone, and sent automatically to the on-line cloud AI server, via wi-fi, along with the participant’s ID. The image analysis server recognized the shape of the palm and the dorsum of both hands from the image, divided each image into 18 sections, for a total of 72, and calculated the percentage of the fluorescently marked ABHR coverage of each section; the covered area as the numerator, and the total area of the divided section as the denominator. The anatomical segmentation is similar to that of a previous study by Ghazali et al. [8], which was validated in their study. Then, a heatmap, colour-coded in five stages based on the uncovered percentage score (0–10, 10–15, 15–23, 23–32, 32–100%) for each section was automatically generated by the AI system within the server; and a quantitative evaluation was obtained. The total coverage (%) for the palm and the dorsum was also automatically calculated as the participant’s “total score”. The total palm/dorsum coverage calculations by the original SCORE! programme included all 72 sections, but for this investigation, the total score was recalculated by excluding 4, the palmar and the dorsum of the wrist section of both hands. Because the programme recognized the ‘wrist’ section as the entire area proximal to the hand that was included in the image, the proximal ends of these sections were not determined anatomically. Therefore, the percentage of the fluorescently marked ABHR coverage of this section could not be obtained with consistency. The heat map images, and the “total score” were displayed on the iPhone screen within 30 s, and the participants were given immediate feedback. The data were saved on the server and were downloaded later for confirmation and analysis of the results.

#### Hand size calculations

Hand surface area (HSA, cm^2^) was measured according to the method previously described by Lee et al. [9]. (HSA = 1.29 × circumference of the metacarpophalangeal joint × hand length.) Using the definitions of hand size presented by Pires et al. [10], the participants’ hand size was categorized as “small” (surface area ≤ 375 cm^2^), “medium” (surface area 376–424 cm^2^), or “large” (surface area ≥ 425 cm^2^). The size of the medical gloves each participant uses in daily practice (small/medium/large), which was referred to in the randomization process, and the dominant hand (right/left), was also recorded.

#### Outcomes

Total palm/dorsum coverage percentage assessments were evaluated by the mean coverage percentage. The coverage of an anatomical section less than 85% was assessed as “insufficient coverage”. The percentage of participants with insufficient coverage was compared in the two techniques.

#### Sample size

Sample size was taken as “all workers of the hospital”, as this study was conducted as part of a regular mandatory training session, to aim for a large-scale study [11].

#### Randomization

Participants were instructed to draw a lot, a small piece of paper with either an A or B written on it, from boxes with the letters S (small), M (medium), or L (large), corresponding to the size of medical gloves they are using on a daily basis. Those with an A subsequently performed the WHO6S technique, while those with a B performed the ASw/ol technique. The same number of lots for A and B was added to each of the boxes, when the lots left in the box became less than a few. No mathematical techniques were used in the randomizing process of this study.

#### Statistical methods

The differences in coverage percentage between WHO6S and A6Sw/oI were tested by Student’s t-test or Welch's t-test for each surface of the hand. One-way analysis of variance and Tukey’s honestly significant difference test were used to evaluate the differences in coverage percentage among the three classifications for the size of the hand surface area by Pires [10]. Pearson's chi-square test or Fisher's exact test was used to assess differences in the percentage of the participants with insufficient coverage (under 85%) for each anatomical section of the hand. Bonferroni adjustment for multiple comparisons was used.

The analysis was undertaken using IBM SPSS version 28 (IBM; Armonk, New York, USA).

#### Trial registration

The study was not registered to any platforms for clinical trials, as it was conducted as a part of a regular mandatory training for the hospital workers in a non-clinical setting.

## Results

### Characteristics of the participants

**Fig. 1:**
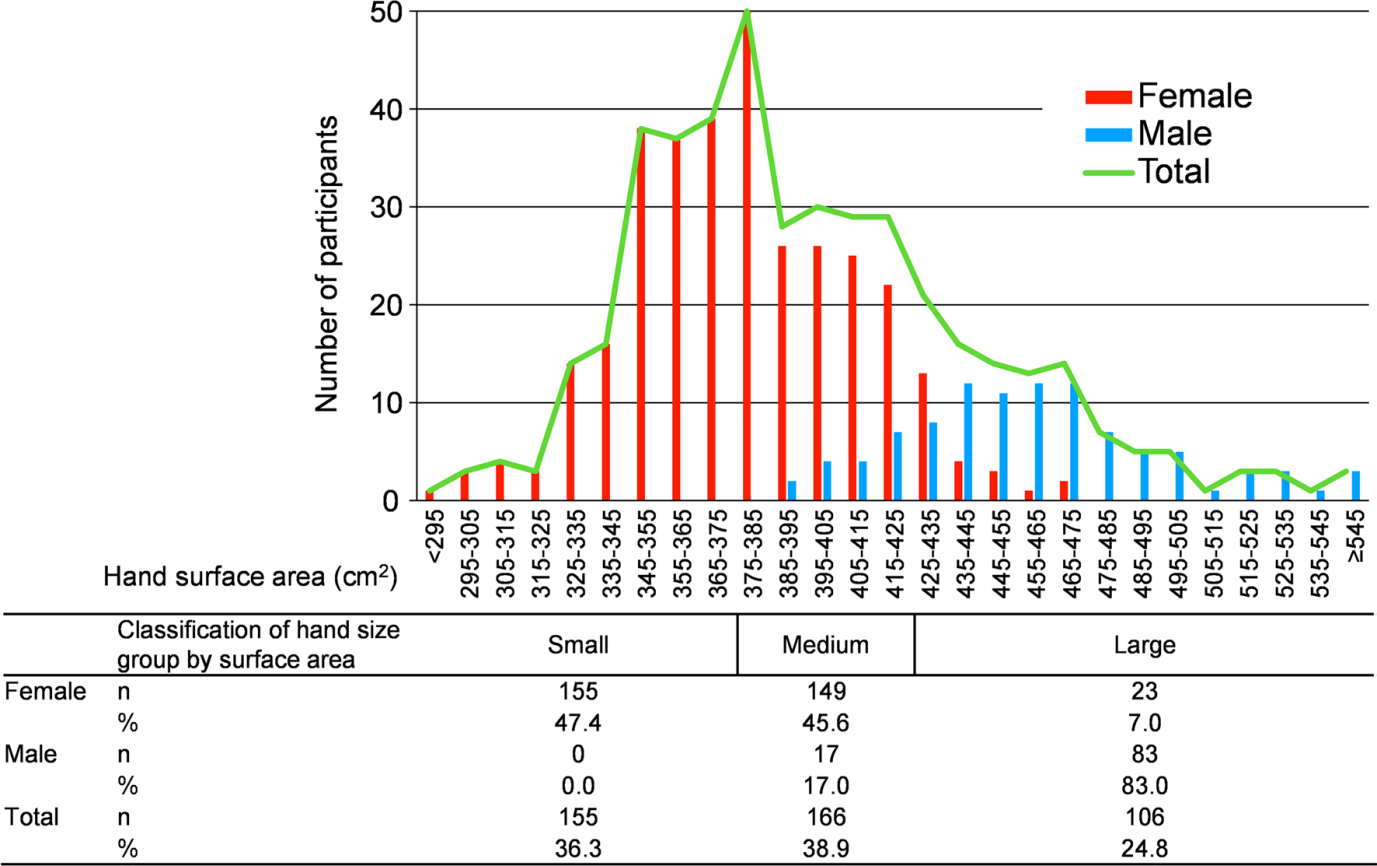
Distribution of hand surface area. Hand surface area calculated by 1.219 × hand length × hand circumference (cm^2^) (Lee et al. [9]). Classification of hand surface area by Pires et al. [10]: small ≤ 375, medium 376–424, large ≥ 425 (cm^2^)

**Fig. 2:**
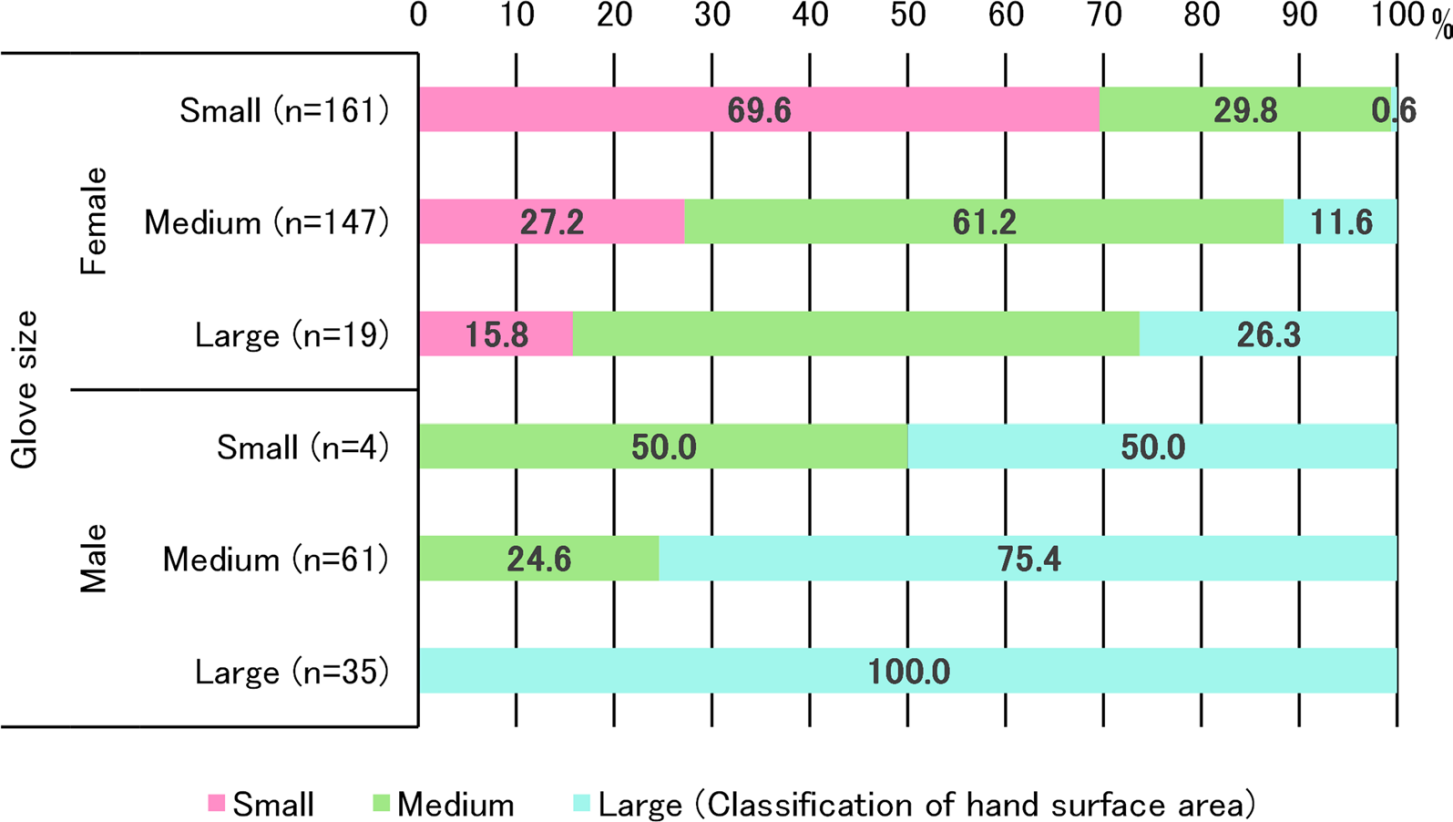
Glove size and classification of hand surface area. Glove size: size of the medical glove used in daily practice, Classification of hand surface area by Pires et al. [10]: small ≤ 375, medium 376–424, large ≥ 425 (cm^2^)

A total of 488 workers were working full-time at the time of the study. Other than the 3 workers with major anomalies and deformations, 26 with severe eczema, and 4 who could not participate for other reasons during the study period, 445 workers were enrolled in the study. After excluding the 18 participants whose data could not be obtained from SCORE! due to tremor of the hands or some other unknown reasons and the 10 participants whose hand size was not measured, data were obtained from 427 participants. A total of 407 participants (95.3%) were right-handed. The number of professions was as follows: 29 physicians, 278 nursing staff, 21 rehabilitation therapists, 26 recreational staff, 97 other co-medical staff, and 41 back-office workers. Details of the characteristics of the participants in the two study groups are shown in Table 1. The WHO6S technique was performed by 215 participants, and the A6Sw/oI technique was performed by 212. The study population in each hand size group was 155 (155 female: 100%) in “small hands” (surface area ≤ 375 cm^2^), 166 (17 male: 10.2% 149 female: 89.8%) in “medium hands” (surface area 376–424 cm^2^), and 106 (83 male: 78.3% 23 female: 6.6%) in “large hands” (surface area ≥ 425 cm^2^). The distribution of the number of participants according to the hand surface area is shown in Fig. 1. The relationship between glove size and classification of hand surface area is shown in Fig. 2. All male participants had medium or large hands, although some of them used small gloves.

**Table 1:** Characteristics of the study participants

	WHO6S		A6Sw/oI	
	n	%	n	%
Total	215	100.0	212	100.0
Sex				
Females	162	75.3	165	77.8
Males	53	24.7	47	22.2
Professions				
Physicians	17	7.9	11	5.2
Nursing staff	131	60.9	133	62.7
Rehabilitation therapists	9	4.2	12	5.7
Recreational staff	14	6.5	12	5.7
Other co-medical staff	24	11.2	25	11.8
Back office workers	20	9.3	19	9.0
Glove size^a^				
Small	83	38.6	82	38.7
Medium	104	48.4	104	49.1
Large	28	13.0	26	12.3
Hand surface area group^b^				
Small	81	37.7	74	34.9
Medium	84	39.1	82	38.7
Large	50	23.3	56	26.4

	Mean	SD	Mean	SD
Hand surface area (cm^2^)				
	397.3	51.4	397.9	45.6

*WHO6S* World Health Organization (WHO) 6-Step, *A6Sw/oI* Adapted 6-Step without Interlock, *SD* standard deviation

^a^Glove size used in daily work

^b^Classification of hand surface area by Pires et al. [10]: small ≤ 375, medium 376–424, large ≥ 425 (cm^2^)

### Mean coverage

Overall mean palm coverage for all hand sizes was 97.8% and 97.5% for the left and right hands, respectively by the WHO6S technique and 97.9% for both hands by the A6Sw/oI technique. Coverage by the A6Sw/oI technique was slightly but statistically higher than the WHO6S in the right hand; this significant difference was seen only in medium hands when assessed by hand size groups (Table 2). Overall mean dorsum coverage varied from 93.7% (right, small hands, WHO6S) to 83.0% (left, large hands, A6Sw/oI). Coverage of more than 90% was obtained for the dorsum of both hands and both techniques by small hands, whereas the coverage for large hands was under 86%. Coverage was higher by the WHO6S in both hands of all hand sizes, and significant differences between the two techniques were found in both small hands and right medium hands (Table 2).

**Table 2:** Coverage area according to hand rubbing techniques

		Palm					Dorsum				
		WHO6S		A6Sw/oI		*p* value	WHO6S		A6Sw/oI		*p* value
		Mean	SD	Mean	SD		Mean	SD	Mean	SD	
All participants	Both	97.9	1.4	98.1	1.7	0.144	90.6	6.9	88.4	8.3	**0.004**
n = 427	Left	97.8	1.5	97.9	2.6	0.845	89.7	7.7	88.0	9.1	**0.032**
	Right	97.5	2.0	97.9	1.5	**0.031**	90.9	7.0	88.4	8.8	**< 0.001**
Small	Both	98.1	1.5	98.4	0.9	0.226	93.6	4.2	91.3	5.8	**0.005**
n = 155	Left	98.2	1.0	98.2	1.1	0.948	93.1	5.0	91.1	6.4	**0.042**
	Right	97.7	2.7	98.1	1.0	0.201	93.7	4.4	90.9	6.2	**0.002**
Medium	Both	97.9	1.2	98.2	2.0	0.296	90.8	6.0	89.1	6.7	0.085
n = 166	Left	97.9	1.3	97.7	3.7	0.707	89.9	6.7	88.5	7.8	0.229
	Right	97.6	1.5	98.3	0.9	**< 0.001**	91.2	6.2	89.2	7.2	**0.046**
Large	Both	97.4	1.5	97.6	2.0	0.544	85.2	8.5	83.6	11.0	0.415
n = 106	Left	97.2	2.1	97.7	1.9	0.186	84.1	9.4	83.0	11.5	0.594
	Right	97.3	1.5	97.1	2.3	0.725	85.9	8.9	83.8	11.7	0.320

*P*-values of bold shows < 0.05

*SD* standard deviation, *p* value: Student t-test or Welch's t-test

Significant coverage differences were found between hand size groups, especially in the dorsum.

Differences in the coverage area of according to hand size.

**Table:** 

			*P* value of one-way ANOVA and Tukey's multiple comparison test			
			Palm		Dorsum	
			WHO6S	A6Sw/oI	WHO6S	A6Sw/oI
Both	One-way ANOVA		**0.015**	**0.039**	**< 0.001**	**< 0.001**
	Tukey's multiple comparison test	Small versus medium	0.630	0.808	**0.010**	0.198
		Small versus large	**0.011**	**0.036**	**< 0.001**	**< 0.001**
		Medium versus large	0.087	0.122	**< 0.001**	**< 0.001**
Left	One-way ANOVA		**< 0.001**	0.445	**< 0.001**	**< 0.001**
	Tukey's multiple comparison test	Small versus medium	0.343	No significant difference by one-way ANOVA	**0.009**	0.134
		Small versus large	**< 0.001**		**< 0.001**	**< 0.001**
		Medium versus large	**0.021**		**< 0.001**	**< 0.001**
Right	One-way ANOVA		0.499	**< 0.001**	**< 0.001**	**< 0.001**
	Tukey's multiple comparison test	Small versus medium	No significant difference by one-way ANOVA	0.803	**0.034**	0.390
		Small versus large		**< 0.001**	**< 0.001**	**< 0.001**
		Medium versus large		**< 0.001**	**< 0.001**	**< 0.001**

*WHO6S* World Health Organization (WHO) 6-Step, *A6Sw/oI* Adapted 6-Step without Interlock

Classification of hand surface area by Pires et al. [10]: small ≤ 375, medium 376–424, large ≥ 425 (cm^2^).

### Insufficient coverage for each anatomical section of the hand

**Fig. 3:**
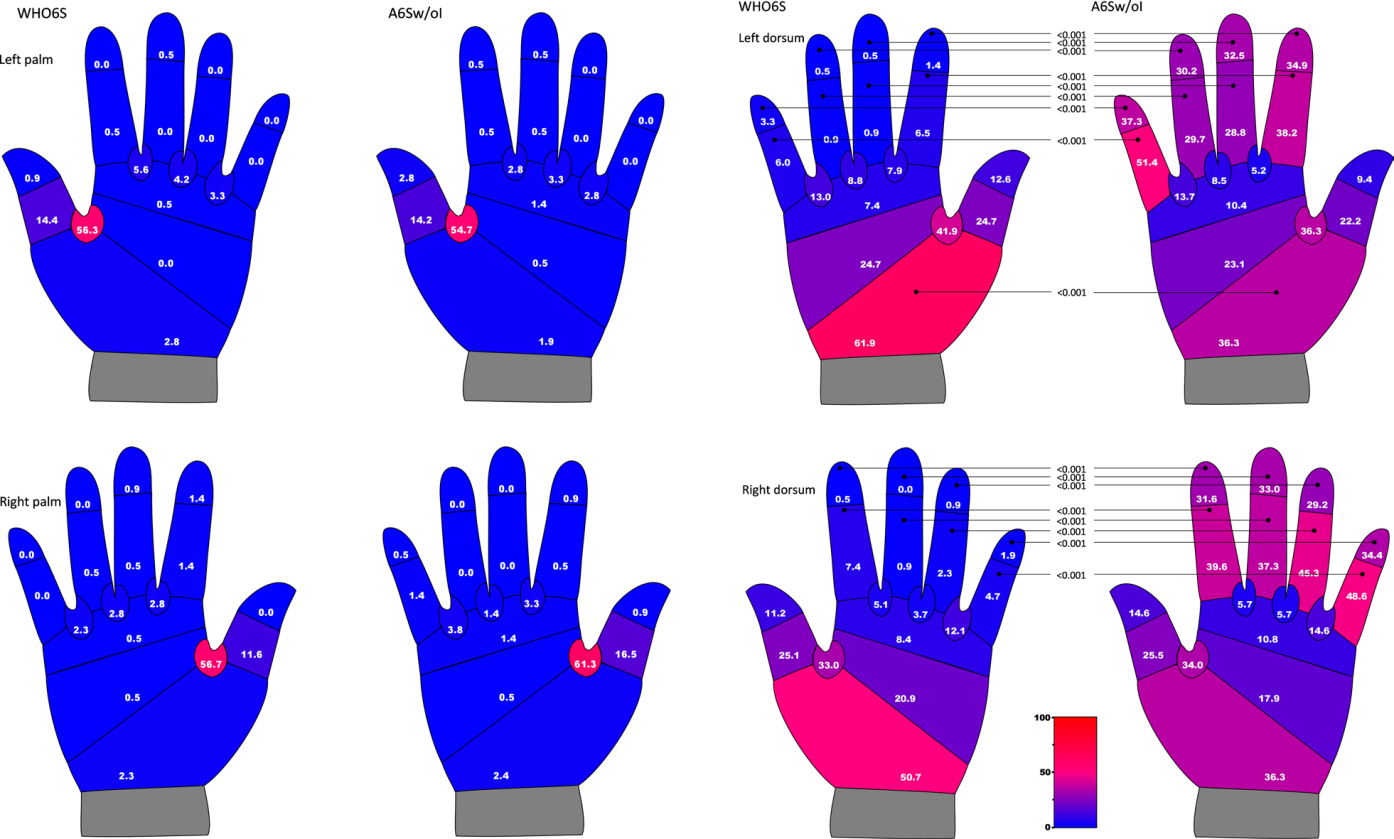
Percentage of participants with insufficient coverage for each anatomical section. *WHO6S* World Health Organization (WHO) 6-Step, *A6Sw/oI* Adapted 6-Step without Interlock. Pearson's chi-square test or Fisher's exact test, *P* values were after Bonferroni correction. Numbers show the percentage of participants with insufficient (under 85%) coverage for each anatomical section

The differences in the percentage of the participants with insufficient coverage for each anatomical section of the hands, including all hand sizes, are shown in Fig. 3. The percentage of the participants with insufficient coverage of the palm were similar for both techniques and hands. In most sections, less than 6% of the participants showed insufficient coverage for both techniques for both hands, except for the thumb-finger web space to the middle part of the thumb. Regarding the four fingers, less than 1.5% of the participants showed insufficient coverage. However, the results of the dorsum showed different, characteristic tendencies. Regarding the backs of the four fingers, the percentage of the participants with insufficient coverage was less than 8% for the WHO6S, whereas for the A6Sw/oI, this was more than 28% for both hands, all significantly higher. This tendency was reversed in the proximal section of the dorsum of the hand, just above the wrist. This section was most often missed by the WHO6S; more than 50% of the participants showed insufficient coverage for both hands. This was lower for the A6Sw/oI group, 36.3% for both hands, and the left hand showed a significant difference.

**Fig. 4:**
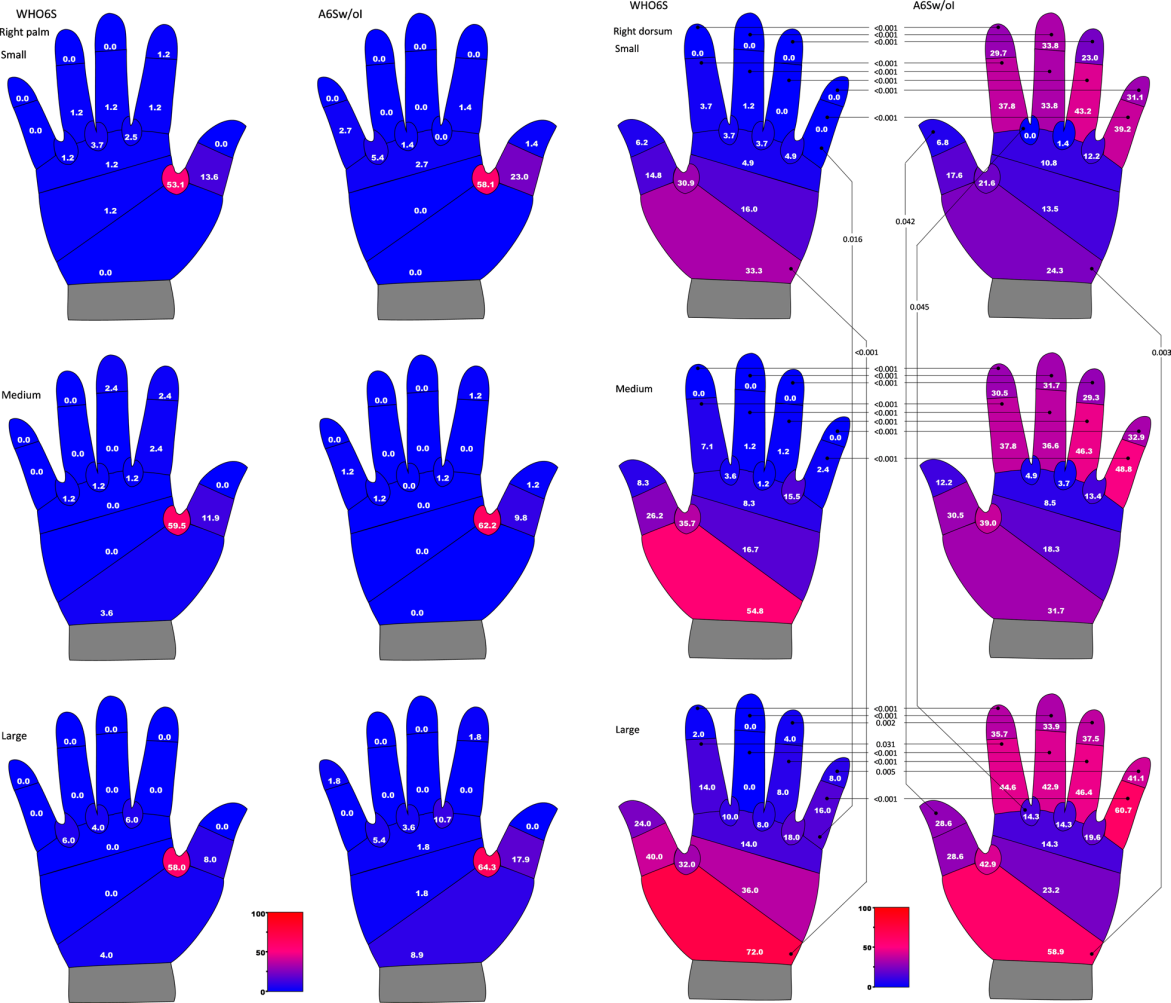
Percentage of participants with insufficient coverage for each anatomical section according to hand size. *WHO6S* World Health Organization (WHO) 6-Step, *A6Sw/oI* Adapted 6-Step without Interlock. Pearson's chi-square test or Fisher's exact test, *P* values were after Bonferroni correction. Classification of hand surface area by Pires et al. [10]: small ≤ 375, medium 376–424, large ≥ 425 (cm^2^). Numbers show the percentage of participants with insufficient (under 85%) coverage for each anatomical section

The differences in the percentage of the participants with insufficient coverage for each anatomical section of the right hand, according to hand size, are shown in Fig. 4. The right hand was chosen because it was the dominant hand for more than 95% of the study population, and hence, it would take more part in transmitting microbes. The results of the palm for both techniques, for all hand sizes, were similar to the results shown in Fig. 3, and no significant differences were found. The results of the dorsum varied between the techniques and the hand sizes. Regarding the backs of the four fingers, the percentage of the participants with insufficient coverage was significantly lower by the WHO6S in all hand sizes. The proximal section of the dorsum of the hand was most likely to be missed by the WHO6S in all hand sizes. Statistical differences between hand size were found between small and large hands in 2 sections for the WHO6S technique and 3 for the A6Sw/oI technique, both including the proximal section of the hand.

## Discussion

The A6Sw/oI technique showed significantly low coverage regarding the backs of the four fingers and the overall mean dorsum coverage for both hands compared to the WHO6S. The coverage of the proximal section of the dorsum of the hand, just above the wrist, was low in both techniques, and was lower in larger sized hands. Only small hands could obtain more than 90% coverage for the dorsum of both hands by the 1.1 ml ABHR-based substance used in this study. The coverage of the palm was generally greater than 90% and was not affected by the difference in techniques or hand size. A significant difference in dorsum coverage was found between small and large sized hands for both techniques.

A technique to cover all hand surfaces was standardized as the WHO6S in the WHO guidelines in hand hygiene [3] in 2009 and is still considered as the world standard up to today. However, some current locally adapted hand rubbing procedure diagrams [4, 6] do not include the “interlock” step. This step tends to be unconsciously ignored in HH practice; there are several studies that show its low adherence in clinical settings. In one study, the “interlock” step was the least observed step of the 6 steps, with the adherence of around 30% by the group that performed the simplified 3 steps, and 75% by the WHO6S group, respectively [12]. Other studies also report that the adherence to this step was as low as 21.5% [13] and 33.2% [14] by the HCWs performing the WHO6S. There is also a study referring to the WHO guidelines that excludes the “interlock” step, as the authors considered that this can be covered by the “palm over dorsum” step [15]. Some other local adaptations add the “wrist” step to the WHO’s 6 steps [16, 17]. However, the evidence for these local adaptations is not clarified. Our study demonstrated that adding the “wrist” step and omitting the “interlock” step, as in A6Sw/oI, resulted in lower overall mean dorsum coverage, and lower coverage of the backs of the fingers, in a structured setting that affirms high adherence to all 6 steps.

The clinical significance of disinfecting the backs of the fingers is not yet clear; however, this surface of the hand is likely to touch body fluid during oral care or changing diapers, touch the patients’ skin in some blood sampling techniques, and may also be contaminated when doffing gloves. Longtin et al. showed that the surface of the hand that gets closer to the patients’ skin during physical examination tended to become more contaminated in their study on the contamination of stethoscopes [18]. The backs of the fingers were not analyzed in their study; however, as they are relatively closer to the patients’ skin, they are much more likely to touch patients and environments frequently than the proximal surface of the dorsum of the hand and/or the wrist.

“Adapt to adopt” is widely recommended for embedding the WHO HH strategy effectively in different areas of the world. However, it is not always as clear as to what extent arrangements and modifications can be regarded as “adaptations”. Careful considerations based on scientific evidence are needed when applying local empirical practice as an adaptation of the WHO guideline.

There are many previous studies on the missed areas of the hands in hand hygiene, and the most-missed areas have often been said to be the thumbs and/or the fingertips [13, 14, 19–22]. There are many factors that affects coverage in hand hygiene; technique, volume-hand size relationship, or adherence, and a mixture of these challenges are present in clinical practice. Many of the previous studies that reported the poor coverage of the thumbs and/or fingertips, may have mainly reflected the low adherence to the step to cover these surfaces. In our study, under a setting with high adherence to all 6 steps, we found that they were well covered. However, we could not assess the tip surfaces of the thumbs and fingers, because the coverage assessment system we used is not based on 3D scanning, and this may have had some effect on the high coverage of the thumbs and fingertips in our study.

The low coverage of the backs of the four fingers by the A6Sw/oI reflects the lack of the step to cover this surface in this technique. However, the low coverage of the dorsum of the hands by the WHO6S was observed, despite the high adherence to the “palm over dorsum” step. This result is consistent with previous studies; Reilly et al. showed that the dorsum of the hands showed the lowest coverage (around 45% of the participants not fully covering the region), while the “palm over dorsum” step showed over 95% adherence [12]. The coverages of the backs of the fingers were relatively higher (participants not fully covering the region: 25% or lower) while the adherence of the “interlock” step was the lowest of the 6 steps by their WHO6S group, around 75%. Interestingly, the coverage of the dorsum of the hands was superior in their 3-step group; participants not fully covering this surface was around 20%, despite the lower adherence (around 55%) to the “palm over dorsum” step. A6Sw/oI group in our study also showed superior coverage of the dorsum of the hands, especially for the section just above the wrist. This can be explained by the unintentional, partial coverage that was observed by the “wrist” step, which is not included in the WHO6S. Significant difference between the techniques was found only in the left hand, which might reflect the fact that over 95% of the participants were right-handed.

Insufficient amount of ABHR applied for the hand size has been discussed as responsible for the poor dorsal coverage in many previous studies [22–24], and we also found that the percentage of the participants with insufficient dorsal coverage increased as the hands were larger. However, if this was the main cause, theoretically, the coverage of the thumbs and the fingertips would be expected to be even lower, because the steps to cover these surfaces are after the “palm over dorsum” step for both techniques. While taking our data, we observed that many participants tended to focus more on rubbing the interdigital web space rather than the dorsal surface of the hand at the “palm over dorsum” step, which follows the findings from the study by Durso et al. that also showed the low coverage of the dorsum by the WHO6S [25]. Some participants, especially the back-office workers, tended to have difficulty placing their hands in the correct position for this step, as it requires relatively large movements of the wrists, in positions that are rarely taken otherwise. A certain proportion of the participants rubbed the dorsum of the hand differently, naturally and unintentionally; instead of placing one hand over the other in the same direction, they placed the hands at right angles, moved the top hand sideways, and rubbed the dorsum of the hand and the fingers in long strokes. Although the interdigital web space was being missed, this seemed to better cover the dorsum of the hand (and the fingers) than the WHO “palm over dorsum” step. The coverage of the interdigital web space may benefit in bacterial reduction in glove juice studies, however, the unnatural positions that require workload may lower adherence in clinical settings. One clinical study suggested that a simpler technique may improve adherence to cover all surfaces and to the five indications of hand hygiene, with similar bacterial log reductions as the WHO6S [14]. As mentioned in a systematic review by Price et al. [26], the WHO6S technique is based on EN1500, the standardized testing method for hand hygiene products [27], which was originally not intended to be performed heavily in everyday clinical practice, and its adherence is low [15, 21]. Pursuing an effective technique to rub the dorsum of the hand, along with applying an adequate amount of ABHR for hand size, may not only contribute to achieving better coverage but may also contribute to higher adherence. Further research is needed to define the most efficient hand rubbing technique in practice, regarding coverage, bacterial reduction, and adherence.

Voniatis et al. reported that 3 ml is a reasonable volume to cover medium-sized hands [22]. Kenters et al. reported that 2.25 ml is needed to cover both sides of the hands adequately (82–90%), although the hand size of the participants was not described, and also that more than 86% of the HCWs used only one push per event regardless of hand size [23]. We also observed similar trends; “one push per event, by everyone”, in our direct observation sessions in clinical practice. Therefore, from this perspective, the volume used in this study reflects current practices in reality. The 1.1 ml ABHR used in our study could obtain a mean coverage of over 90% for both sides by the workers with small hands, and their hands were generally observed to be still fairly wet at the image taking process. Therefore, for this group, 3 ml, the amount fixed in previous studies in clinical settings [12, 14] may be unfeasible, with drippings on the floor and the length of time it would take to dry. On the other hand, large hands were generally observed to be completely dry by the end of the 25 s of the hand rubbing process. Over 60% of all participants and 100% of the male participants had medium to large hands, which suggests the possibility that the majority of the workers in our hospital are not using enough ABHR for their hand size. We give the healthcare workers a choice between different high-quality hand rubs to carry personally, to ensure maximum acceptability, as in [28], and the one push volume of the ABHR products available in our hospital varies from 1.1 to 1.8 ml. However, the workers choose the products they use based on their tastes, such as texture and moisturization to the skin, and not the volume per push. The amount of ABHR needs to be customized according to hand size [29, 30], and we need to train workers with larger hands to use 2 or 3 pushes, depending on their hand size and the product they choose. Personal carriable dispensers with controllable ‘volume per push’ might be helpful, to improve adherence to use the optimal amount for each individual.

Recently, studies based on “fluorescent dye-based hand rubbing quality assessment”, which was validated by Lehotzky et al. with microbiological assessments [31], have been increasing. Additionally, showing the missed areas by heatmaps is visually informative when assessing the coverage of the hand. Some such studies have compared the differences and relation between the amount and/or the hand size [22, 32]. Another recent study visualized the difference of the missed areas between the rubbing techniques, but both the amount of the substance used, and the hand size of the participants were not described [25]. Our study is the first to quantitatively evaluate the difference in the coverage of the regions of the hands, visualized by heatmaps, between two different hand rubbing techniques, with the description of both the amount of ABHR and the hand size of the participants.

As stated in a recent systematic review, HH research should consider ABHR volume, application time, and hand size [33]. Our study followed this by standardizing both the volume and the application time, and considering hand size by applying the size of the medical gloves in the randomization process to minimize the hand size differences between the two techniques. Significant differences in coverage between hand sizes within both technique groups were found; therefore, studies comparing techniques without descriptions of both the ABHR volume and hand size, may reflect differences in volume-hand size relationship between the groups. There is a growing consensus that volume should ideally be customized [30], and this should be considered in future research on hand hygiene techniques [33].

### Limitations

The UV marked substance that was used to ‘hand rub’ had lower alcohol concentration and higher viscosity compared to the original ABHR product. The drying time and texture felt acceptable, the difference within the variation between products; however, quantitative data on the volatility and the viscosity of the substance was not taken and may have had some effect on the results. Also, the process to “rub until hands are dry”, that are originally included in the total procedure of hand rubbing by WHO6S technique, were omitted. From these points of view, drying time was not assessed in this study, although it is one of the important factors that influences efficacy.

The structured environment in which the study was conducted, likely yielded results that do not reflect what happens during routine patient care, and difference in adherence may affect the results in clinical settings.

The lack of training may have affected the results. This was the first mandatory technique training session for all hospital workers, with visual feedback taught directly by the Infection Control Nurse. Better results may have been achieved if such training had been continued.

The lateral surfaces of the hands, including surfaces of the tips of the thumbs and fingers were not analyzed, as the device used in this study is not based on 3D scanning, and the coverage ratio of the 360° hand surface area was not evaluated. Therefore, especially for the participants with thick hands, total hand coverage and the coverage of the sections that are adjacent to the lateral surfaces, may be much lower.

Microbiological assessments were not performed in this study, and there is no data on the bacterial log reductions achieved by the two techniques.

## Conclusion

The WHO 6-step technique was superior to a locally adapted 6-step technique without interlock regarding dorsum surface coverage, especially for the backs of the four fingers. Careful consideration is necessary when applying locally practiced techniques as adaptations of guidelines. The volume- hand size relationship should be considered when assessing coverage differences between hand rubbing techniques.

## Supplementary Information

**Additional file 1.** (A) WHO6S diagram.

**Additional file 2.** (B) A6Sw/oI diagram.

## Data Availability

Available from the corresponding author on reasonable request.

## Abbreviations

A6Sw/oI: Adapted 6-step without interlock technique
ABHR: Alcohol-based hand rub
AI: Artificial intelligence
HH: Hand hygiene
HAS: Hand surface area
HCWs: Health care workers
UV: Ultraviolet
WHO: World Health Organization
WHO6S: World Health Organization 6-step technique
