# Correction: Comparison of two alcohol hand rubbing techniques regarding hand surface coverage among hospital workers: a quasi-randomized controlled trial

**DOI:** 10.1186/s13756-022-01195-8

**Published:** 2022-12-17

**Authors:** Yumi Suzuki, Motoko Morino, Ichizo Morita, Sumie Ohiro

**Affiliations:** 1 Department of Pediatrics, NHO Shimoshizu National Hospital, 934-5 Shikawatashi, Yotsukaidou-City, Chiba 284-0003 Japan; 2 Division of Infection Control, NHO Shimoshizu National Hospital, Yotsukaidou, Japan; 3 Department of Nursing, NHO Shimoshizu National Hospital, Yotsukaidou, Japan; 4 Japanese Red Cross Toyota College of Nursing, Toyota, Japan

**Correction to: Antimicrobial Resistance & Infection Control (2022) 11:132** 10.1186/s13756-022-01172-1

The original article [1] contained errors in Table 2’s formatting which have since been corrected.

These errors were mistakenly carried forward by the production team handling the article, and thus were not the fault of the author.
