# Supplementary figures and images for: Comparison of two alcohol hand rubbing techniques regarding hand surface coverage among hospital workers: a quasi-randomized controlled trial
Source: Antimicrob Resist Infect Control. 2022 Nov 3;11:132. doi: 10.1186/s13756-022-01172-1 (PMC9635155; doi:10.1186/s13756-022-01172-1)

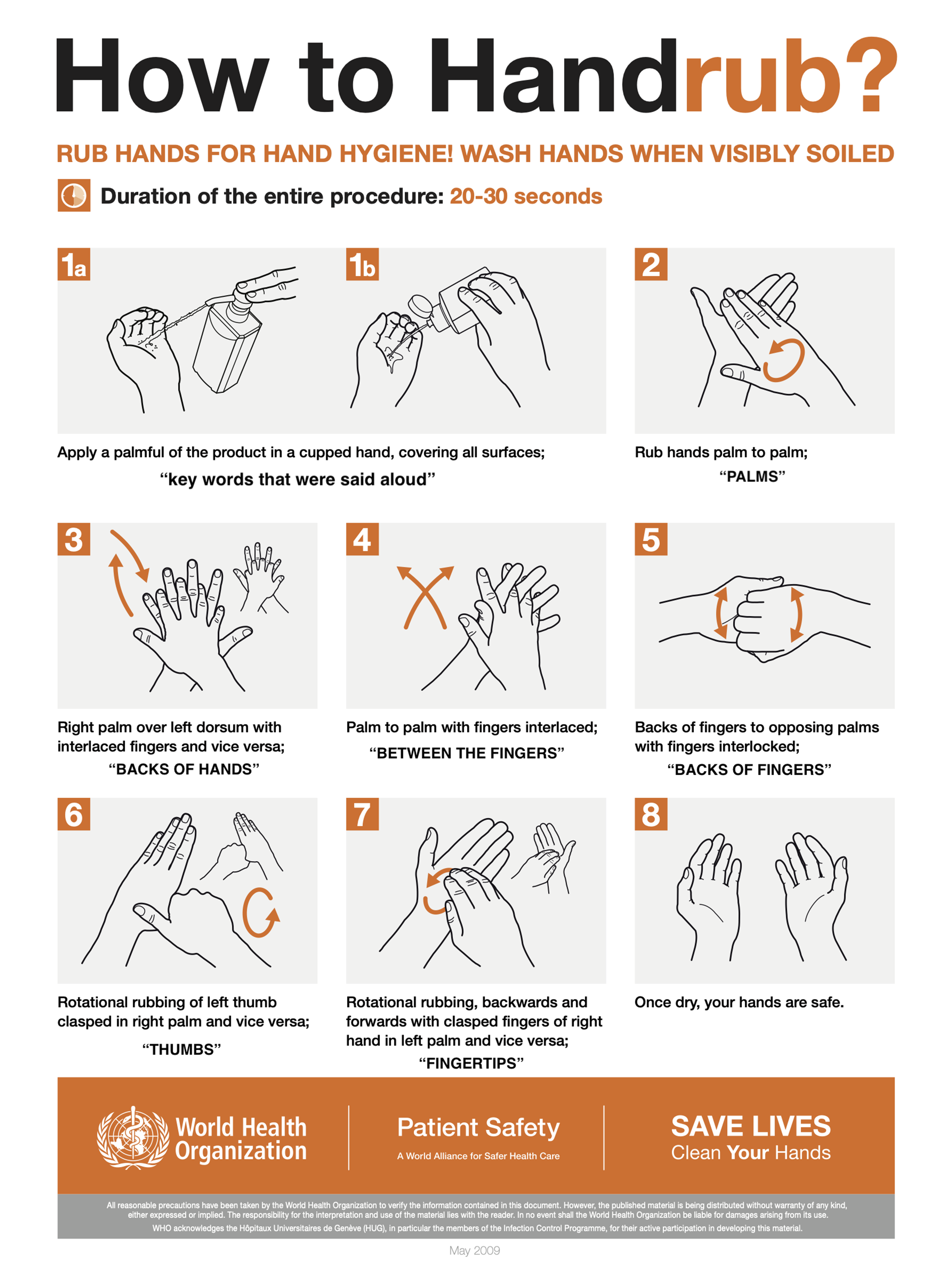

Supplement: Supplementary file 1 — Additional file 1. (A) WHO6S diagram. [file 13756_2022_1172_MOESM1_ESM.tif]

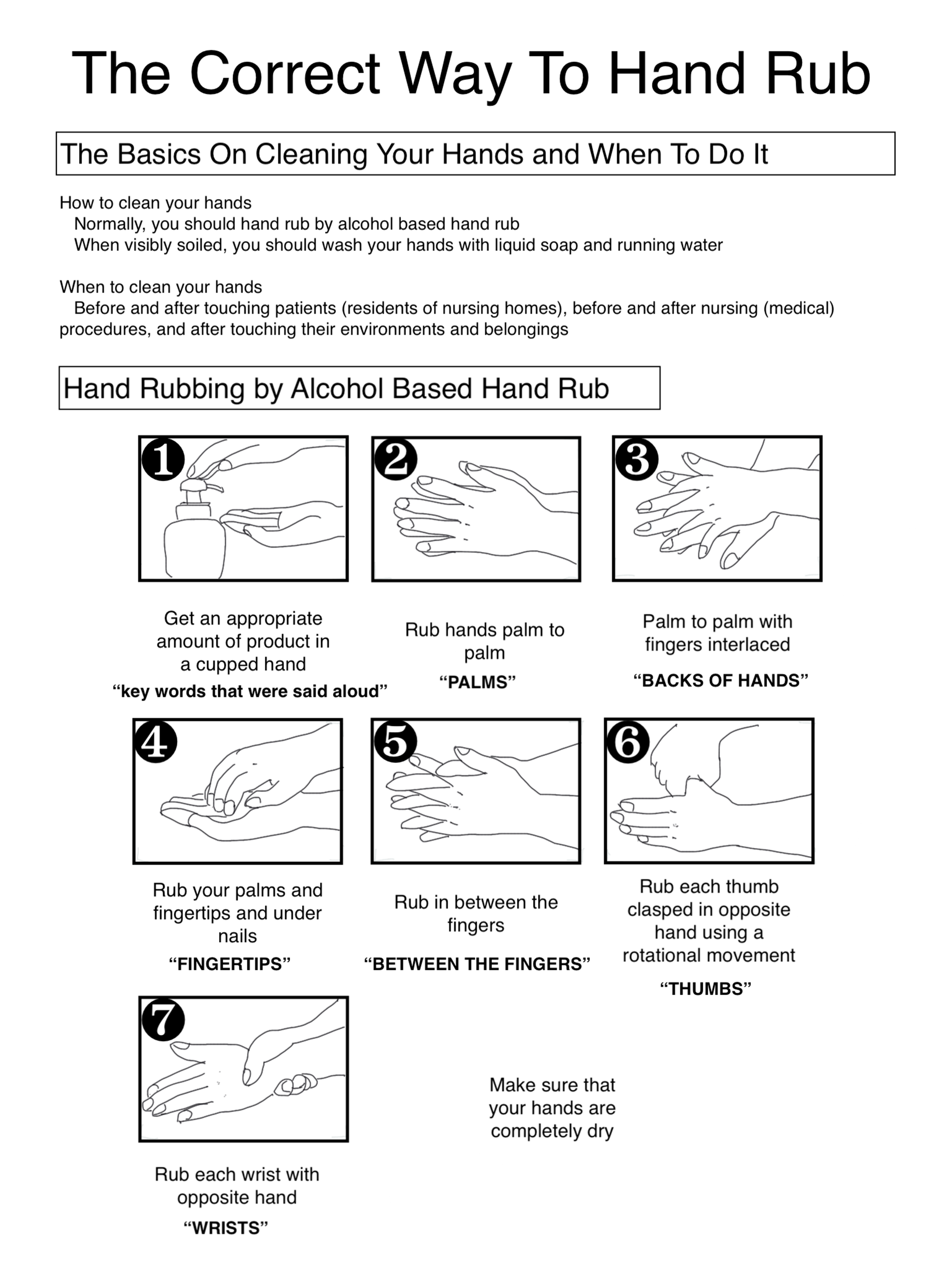

Supplement: Supplementary file 2 — Additional file 2. (B) A6Sw/oI diagram. [file 13756_2022_1172_MOESM2_ESM.tif]
